# Nano-Enabled Delivery of Phage-Based Antibacterials Against ESKAPE Pathogens

**DOI:** 10.3390/pharmaceutics18020185

**Published:** 2026-01-30

**Authors:** Ayman Elbehiry, Eman Marzouk, Adil Abalkhail

**Affiliations:** Department of Public Health, College of Applied Medical Sciences, Qassim University, P.O. Box 6666, Buraydah 51452, Saudi Arabia; ar.elbehiry@qu.edu.sa (A.E.); e.marzouk@qu.edu.sa (E.M.)

**Keywords:** antibiotic resistance, ESKAPE pathogens, bacteriophages, phage-derived enzymes, nanotechnology-based delivery, nanocarrier, CRISPR antimicrobials, biofilm-associated infections

## Abstract

Antimicrobial resistance (AMR) remains a major clinical challenge, with *Enterococcus faecium*, *Staphylococcus aureus*, *Klebsiella pneumoniae*, *Acinetobacter baumannii*, *Pseudomonas aeruginosa*, and *Enterobacter* species (ESKAPE) accounting for a substantial share of multidrug-resistant (MDR) infections worldwide. These organisms undermine antibiotic efficacy through reduced permeability, surface shielding, biofilm formation, and rapid genetic adaptation, mechanisms that primarily restrict effective exposure at infection sites. Bacteriophages, phage-derived enzymes, and Clustered Regularly Interspaced Short Palindromic Repeats (CRISPR)-based antimicrobials provide selective and mechanistically distinct alternatives to conventional antibiotics, but their performance in vivo is often limited by instability in physiological environments, immune neutralization, uneven tissue distribution, and insufficient access to bacteria protected by biofilms or surface-associated barriers. This narrative review examines how nanotechnology-based delivery systems can address these constraints. We first outline the delivery-relevant biological barrier characteristic of ESKAPE pathogens, then summarize the therapeutic potential and inherent limitations of whole phages, phage-derived enzymes, and CRISPR-based antimicrobials when used without formulation. Major nanotechnology platforms for antibacterial delivery are reviewed, followed by analysis of how nano-enabled systems can improve stability, localization, and persistence of these biological agents. A pathogen-aware integration framework is presented that links dominant barriers in each ESKAPE pathogen to the biological modality and nano-enabled delivery strategy most likely to enhance exposure at infection sites. Translational challenges, regulatory considerations, and emerging directions, including responsive delivery systems and personalized approaches, are also discussed. Overall, nano-enabled phage-based therapeutics represent a realistic and adaptable strategy for managing MDR ESKAPE infections. Therapeutic success depends on both continued discovery and engineering of antibacterial agents and effective delivery design.

## 1. Introduction

Antimicrobial resistance (AMR) is a major global health threat. In 2019, AMR was estimated to have directly caused approximately 1.27 million deaths worldwide and to have contributed to nearly 4.95 million deaths overall [1,2]. Global surveillance data continue to show rising resistance rates, reinforcing the need for alternative therapeutic strategies [3]. The World Health Organization identifies AMR as a critical public health challenge, with projections indicating a substantial increase in mortality by 2050 in the absence of effective interventions [2]. A group of clinically important bacteria known as ESKAPE pathogens includes *Enterococcus faecium* (*E. faecium*), *Staphylococcus aureus* (*S. aureus*), *Klebsiella pneumoniae* (*K. pneumoniae*), *Acinetobacter baumannii* (*A. baumannii*), *Pseudomonas aeruginosa* (*P. aeruginosa*), and *Enterobacter* species. These organisms are frequent causes of hospital-acquired infections and often exhibit resistance to multiple antibiotics [4,5]. They account for a substantial proportion of healthcare-associated AMR because they readily accumulate and exchange diverse resistance determinants.

Conventional antibiotics often fail against ESKAPE pathogens because of biological barriers that limit effective drug exposure. Many strains form dense biofilms or produce protective capsules that restrict antibiotic penetration [6]. In parallel, resistance mechanisms such as β-lactamases, efflux pumps, and target-altering mutations further reduce antibiotic efficacy [7]. These features limit the utility of even last-line antibiotics [8].

Biological antibacterials offer mechanistically distinct alternatives to conventional antibiotics. These include whole bacteriophages, phage-derived enzymes such as lysins and depolymerases, and CRISPR-based antimicrobial [9,10]. Their high specificity can preserve commensal microbiota, but their performance in vivo is often constrained by limited stability, immune neutralization, uneven tissue distribution, and poor access to bacteria protected by biofilms or surface-associated barriers [11]. These limitations primarily reflect delivery and exposure challenges rather than insufficient intrinsic antibacterial activity. As a result, effective therapeutic use often requires delivery systems that protect biological agents, localize them to infection sites, and sustain activity within complex infection environments [12].

Nanotechnology provides practical tools to address these delivery constraints. Nanocarriers can protect sensitive biological cargos, control release profiles, and improve tissue or mucosal delivery, including access to bacteria embedded within biofilms or shielded by capsules [13]. Encapsulation strategies have been shown to improve the stability and performance of bacteriophages and phage-derived enzymes in experimental models [14]. Recent studies report increasing success with nanoparticle platforms that extend half-life, reduce inactivation during processing or administration, and support targeted or pulmonary delivery [15]. Clinical and compassionate-use experiences further support the therapeutic potential of phage-based treatments when paired with advanced delivery systems [16].

This review follows a convergent framework. It first outlines the biological delivery barriers presented by ESKAPE pathogens. It then summarizes the biology and therapeutic potential of phages and phage-derived antibacterials. Finally, it links nanotechnology-based delivery strategies to pathogen-specific challenges [5,17]. While recent reviews have examined individual modalities such as phage nanodelivery systems, phage-derived enzymes, depolymerases, or CRISPR-based antimicrobials often in isolation, this review integrates pathogen-specific biological barriers with coordinated selection of antibacterial modality and nano-enabled delivery strategy. This delivery-centered, translational perspective distinguishes the present review from prior descriptive or modality-focused approaches.

Accordingly, this review has three objectives. First, it summarizes the key biological barriers that limit treatment of ESKAPE pathogen infections. Second, it analyzes whole phages, phage-derived enzymes, and CRISPR-based antimicrobials in relation to these barriers. Third, it presents a pathogen-aware framework that links antibacterial modality choice with nano-enabled delivery strategies to improve exposure at infection sites.

The literature was selected to support a delivery-focused narrative analysis of phage-based and phage-derived antibacterial strategies against ESKAPE pathogens. Searches were conducted using PubMed, Web of Science, and Scopus, with emphasis on peer-reviewed experimental, translational, and regulatory sources published mainly within the last fifteen years. This review does not aim to provide a systematic or exhaustive survey but instead focuses on representative examples that illustrate how pathogen-specific biological barriers interact with biological antibacterial modalities and nano-enabled delivery strategies.

**Figure 1:**
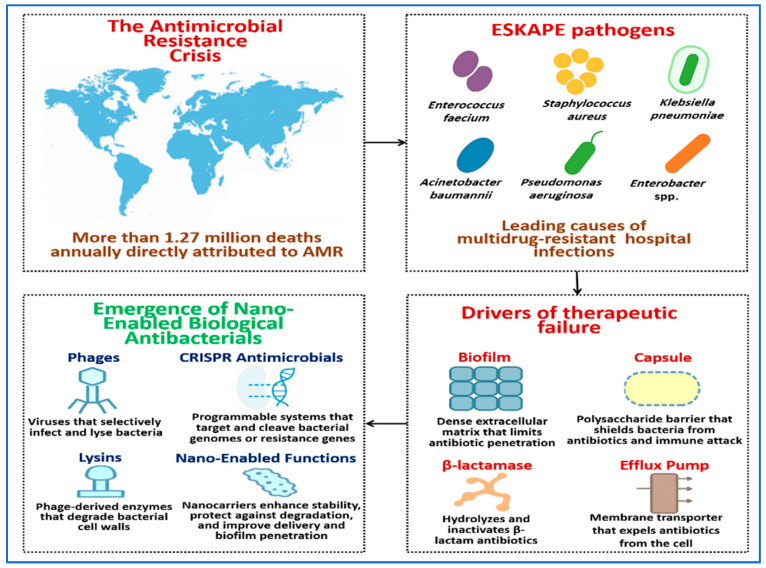
Overview of AMR, ESKAPE pathogens, major drivers of therapeutic failure, and the rationale for nano-enabled biological antibacterials. AMR is responsible for more than 1.27 million deaths annually and is driven in part by ESKAPE pathogens, which are leading causes of MDR hospital-acquired infections. These pathogens persist due to biological barriers that limit antibiotic effectiveness, including biofilm formation, capsular polysaccharides, β lactamase production, and efflux pump activity. Biological antibacterials such as bacteriophages, phage-derived enzymes, and CRISPR-based antimicrobials provide alternative mechanisms of action. Nanocarriers can enhance the stability, localization, and delivery of these agents and improve access to bacteria protected within biofilms or surface-associated barriers.

Figure 1 provides a conceptual overview of the AMR challenge and highlights why ESKAPE pathogens present persistent treatment difficulties. It also introduces nano-enabled phage-based therapeutics as a delivery-focused strategy, framing the central premise of this review that effective exposure at infection sites is a dominant determinant of therapeutic outcome.

## 2. Delivery-Relevant Biological Barriers in ESKAPE Pathogens

Successful antibacterial delivery depends on the physical and biological features present at infection sites. ESKAPE pathogens differ markedly in cell envelope organization, surface structures, biofilm formation, and immune evasion traits. These differences strongly influence how therapeutic agents reach bacterial cells, persist in hostile microenvironments, and maintain activity during infection [15].

This section provides the primary and comprehensive framework for delivery-relevant biological barriers in ESKAPE pathogens. Subsequent sections refer back to these barriers and focus on modality-specific, formulation-specific, or translational implications rather than re-describing the underlying constraints. Understanding these barriers is essential for rational selection of biological antibacterials and compatible delivery strategies, as discussed in later sections.

### 2.1. Cell Envelope Diversity and Permeability Limits

The bacterial cell envelope represents the first major barrier encountered by antibacterial agents. Its structure differs substantially between Gram-negative and Gram-positive ESKAPE pathogens. Gram-negative species, including *K. pneumoniae*, *A. baumannii*, *P. aeruginosa*, and *Enterobacter* species, possess an outer membrane that restricts entry of many antibiotics and large molecules. The outer leaflet contains lipopolysaccharide or lipooligosaccharide, which reduces permeability and limits passive diffusion across the envelope [18].

Entry into Gram-negative bacteria often depends on porin channels. Reduced porin expression or porin loss decreases permeability and is frequently associated with carbapenem resistance in Enterobacteriaceae, including the *Enterobacter cloacae* complex [19,20]. As a result, achieving sufficient intracellular exposure remains a major challenge even when high extracellular drug concentrations are present.

*P. aeruginosa* exhibits particularly low outer membrane permeability and often combines this trait with additional defenses such as efflux systems and biofilm growth. These features markedly reduce effective exposure of antibacterial agents at the bacterial surface [21]. In *A. baumannii*, major outer membrane proteins such as *OmpA* contribute to host interaction, virulence, and biofilm-associated behavior. This envelope architecture can limit both access to the cell surface and retention of antibacterial agents in the local environment [22].

Gram-positive pathogens such as *S. aureus* and *E. faecium* lack an outer membrane but possess a thick peptidoglycan layer decorated with surface polymers. These structures influence surface charge, binding interactions, and susceptibility to antibacterial agents. Enterococci also display strong envelope stress responses and intrinsic resistance traits that support survival under antimicrobial pressure, which can reduce effective killing even when therapeutic exposure occurs [23,24].

### 2.2. Capsules and Surface Polysaccharides That Block Access

Capsules represent an additional surface barrier in several ESKAPE pathogens. Capsular polysaccharides form a hydrated layer that physically shields the bacterial surface, reduces binding of immune factors, and slows diffusion of antibacterial agents toward the cell envelope [25].

*K. pneumoniae* is a prominent example, as its capsular polysaccharide is a major virulence determinant. Capsule composition varies widely among strains, resulting in high antigenic diversity. This variability complicates therapeutic approaches that rely on consistent surface recognition or binding [26]. Capsules and related surface layers are also relevant in *A. baumannii*, where encapsulation and surface remodeling contribute to persistence in hostile host environments and reduced susceptibility to antibacterial interventions [27].

### 2.3. Biofilm-Associated Barriers to Antibacterial Delivery

Biofilm formation is a shared and clinically important trait among ESKAPE pathogens. Biofilms consist of bacterial communities embedded within a self-produced extracellular matrix that adheres to biotic or abiotic surfaces. This matrix restricts penetration of antibacterial agents and creates microenvironments with altered nutrient availability, oxygen tension, and growth rates. These conditions promote high tolerance to therapy even in the absence of new resistance mutations [28].

Across species, biofilm matrices commonly contain polysaccharides, proteins, and extracellular DNA, although their relative abundance varies with organism and growth conditions [29]. In *P. aeruginosa*, the exopolysaccharides Pel, Psl, and alginate play central roles in shaping biofilm architecture and tolerance, particularly in chronic infection settings [30,31].

*K. pneumoniae* readily forms biofilms on both abiotic and biotic surfaces, and this phenotype is frequently associated with persistent infections and limited therapeutic success [32]. Biofilm formation in *A. baumannii* is regulated by multiple pathways, including quorum-linked systems, and is strongly associated with persistence and antibiotic tolerance in hospital environments [33].

In Gram-positive pathogens, *S. aureus* biofilms can be polysaccharide-rich, protein-rich, or mixed, depending on strain and growth conditions. Extracellular DNA is a key structural component in many *S. aureus* biofilms and contributes to matrix integrity and cohesion [34]. Enterococci also form biofilms in clinical contexts such as device-associated and urinary tract infections. In *E. faecium*, surface proteins including *Esp* contribute to biofilm formation in hospital-adapted lineages, and biofilm growth is linked to increased tolerance through limited penetration and matrix protection [35].

### 2.4. Immune Evasion Traits Beyond Biofilm Protection

In addition to biofilm-mediated shielding, ESKAPE pathogens employ immune evasion strategies that influence exposure to antibacterial agents. Capsules can reduce complement activation and phagocytosis, allowing bacteria to persist within host tissues and narrowing the effective window for therapeutic intervention [25]. Biofilms further impair immune clearance by limiting access of immune effectors to embedded cells. This protection can promote chronic inflammation without effective bacterial elimination, contributing to long-lasting infections that are difficult to eradicate using standard treatments [29].

### 2.5. Surface Receptor Variability and Masking

Many antibacterial strategies depend on interactions with surface features such as outer membrane proteins, teichoic acids, capsules, or biofilm matrix components. In ESKAPE pathogens, these features can vary substantially between strains and may change during the course of infection. Capsule diversity in *K. pneumoniae* is a clear example of surface heterogeneity that complicates consistent targeting across clinical isolates [25].

In Gram-negative bacteria, changes in porin expression further alter surface accessibility and permeability. Such changes can reduce effective exposure even when adequate drug concentrations are achieved at the tissue level [19].

### 2.6. Enzymatic Degradation and Hostile Infection Microenvironments

Infection sites often contain bacterial enzymes that degrade therapeutic agents or alter the local environment. *P. aeruginosa* secretes multiple proteases, including elastase and alkaline protease, which can degrade proteins in the surrounding milieu and reduce the stability of protein-based therapeutics [36].

*S. aureus* produces micrococcal nuclease, which degrades extracellular DNA and can reshape biofilm structure over time. By altering the matrix, this activity can change how antibacterial agents move within the biofilm environment [37]. *A. baumannii* encodes virulence-associated enzymes such as phospholipases that contribute to host damage and bacterial survival. Enzyme-rich infection environments therefore present an additional challenge for maintaining the stability and activity of sensitive biological cargos [38].

### 2.7. Pathogen-Specific Integration of Delivery Barriers

The combination of delivery barriers differs across ESKAPE pathogens, and no single delivery concept is universally effective. *E. faecium* and *S. aureus* are characterized by thick Gram-positive cell walls and biofilm matrices that often contain proteins and extracellular DNA [23,35]. *K. pneumoniae* adds a prominent capsule and strong biofilm-forming capacity, with marked capsule diversity across strains [25,39].

*A. baumannii* and *P. aeruginosa* combine Gram-negative envelope barriers with robust biofilm programs and high stress tolerance. These traits restrict access to bacterial cells and reduce persistence of antibacterial agents at infection sites [22]. *Enterobacter* species frequently reduce permeability through porin alterations, which strongly affects entry of many therapeutic agents [19].

These pathogen-specific barrier profiles serve as the reference framework for subsequent sections, which analyze how phages, phage-derived enzymes, CRISPR-based systems, and nano-enabled delivery platforms address these constraints without re-defining them. These contrasts highlight the need for pathogen-aware delivery strategies. They also provide the biological foundation for matching specific biological modalities and nano-enabled delivery approaches to individual ESKAPE pathogens, as developed in the following sections [20].

## 3. Phages and Phage Derived Antibacterials

Bacteriophages are viruses that infect bacteria. Therapeutic applications focus on lytic phages and on phage-derived proteins that damage bacterial surfaces. These biological agents differ from antibiotics in both target recognition and killing mechanisms. They can also be designed to act with high strain or species selectivity, which is particularly relevant for infections caused by ESKAPE pathogens [9].

### 3.1. Lytic Phage Infection Cycle and Therapeutic Relevance

The lytic phage infection cycle proceeds through a defined sequence of events. The phage first adsorbs to a specific bacterial surface receptor. It then injects its genome into the host cell. Viral genes are expressed and replicated using host machinery, followed by assembly of new virions. The cycle ends with host cell lysis and release of progeny phages [40]. This biology underpins two features that are central to therapeutic use. Bacterial killing occurs only in susceptible cells that support phage infection. In addition, local phage amplification can occur where the target bacterium is present. When conditions permit, this amplification can increase antibacterial pressure directly at the infection site [41].

### 3.2. Engineered and Programmable Phages

Natural phages often exhibit a narrow host range because adsorption depends on specific interactions between phage receptor-binding proteins and bacterial surface receptors. As a result, host range modification has become a major focus of phage engineering. Reviews and experimental studies describe expansion of host range through structure-guided and genetic approaches that modify adsorption modules, including tail fibers and related proteins [42,43].

A second engineering strategy is the introduction of additional functions that enhance antibacterial performance. These include antibacterial proteins and gene-editing modules encoded within phage genomes or delivered by phage-derived particles. Such designs aim to add a second mode of action beyond native lysis or to modulate bacterial responses during infection [44,45].

Advances in phage genome editing have further enabled these approaches. CRISPR-assisted methods are widely used to generate phage variants with defined insertions or deletions. These tools support rational design workflows and improve control over phage genetic content [46].

### 3.3. Phage Derived Lysins

Lysins are phage-encoded enzymes that cleave peptidoglycan. During the native lytic cycle, they contribute to host cell lysis at the final stage of phage replication. When produced as purified proteins, lysins can also lyse bacteria from the outside by directly damaging the cell wall. This activity is most pronounced in Gram-positive bacteria, where peptidoglycan is readily accessible [47,48].

In Gram-negative bacteria, the outer membrane restricts access of unmodified lysins to the peptidoglycan layer. This barrier reduces activity but does not eliminate therapeutic interest. Multiple strategies have been explored to enable lysin action against Gram-negative targets, including structural modification and combination approaches [49,50].

Lysin specificity is often broader than that of whole phages. Many studies report activity at the genus or species level rather than strict strain restriction, although this varies depending on the enzyme and bacterial target [48].

### 3.4. Phage Encoded Depolymerases

Many clinically important bacteria produce capsules or other surface polysaccharides that protect the cell surface and support persistence. Some phages encode depolymerases, which are often located on tail-associated structures and degrade these polysaccharides. This activity facilitates access to the bacterial surface and weakens capsule-mediated defenses [51,52].

In *K. pneumoniae*, multiple studies describe depolymerases with strong capsule type specificity. Experimental work shows that these enzymes can digest capsular polysaccharide, reduce biofilm formation, and disrupt established biofilms in strain-matched settings [53].

Depolymerases are therefore positioned as antivirulence antibacterials that disarm key protective structures rather than directly killing cells. Their performance is tightly linked to polysaccharide type, making capsule diversity a major constraint on coverage across clinical isolates [51].

### 3.5. Tailocins and R-Type Pyocins

Tailocins are phage tail-like bacteriocins produced by bacteria. They resemble headless phage tails and kill closely related bacteria through receptor-dependent binding and membrane damage. Their narrow target range is largely determined by receptor-binding proteins [54].

R-type pyocins produced by *P. aeruginosa* are a well-studied example. These particles kill target cells after binding to specific surface receptors, and multiple studies have characterized their receptor specificity and bactericidal spectra [55,56].

Because tailocin activity depends on surface recognition, receptor variability can strongly influence activity across clinical isolates. Engineering of receptor-binding proteins has been discussed as one strategy to retarget these particles and expand coverage [54].

### 3.6. CRISPR Phage Antimicrobials and Phage-Delivered CRISPR Systems

CRISPR-based antimicrobials aim to kill bacteria or remove resistance determinants through sequence-specific targeting of nucleic acids. Early studies demonstrated RNA-guided nucleases delivered by phage-based systems, establishing a framework for precision antibacterial activity and selective pressure within mixed populations [57,58].

Phages have also been engineered to deliver CRISPR systems that target antibiotic resistance plasmids. In widely cited work, both temperate and lytic phages were programmed to deliver CRISPR constructs that destroyed resistance-conferring plasmids, supporting plasmid curing as a therapeutic strategy [59,60].

CRISPR systems can also be designed for direct bactericidal activity. Cas13a-based antibacterial capsids have been reported to induce sequence-specific killing after recognition of targeted genes, and this approach has been developed as a programmable antimicrobial platform [61].

More recent studies have expanded delivery formats. Non-replicative phage particles and phagemid systems have been used to deliver CRISPR cargo targeting β lactamase genes in model organisms. These strategies aim to promote resensitization or apply selective pressure against resistant subpopulations [32,62].

### 3.7. Limitations of Unformulated Phage-Based Antibacterials

Evidence supporting whole-phage efficacy is strongest at the experimental and compassionate-use level [16]. Controlled clinical trial data remain limited and have highlighted variability in exposure, susceptibility dynamics, and delivered active dose [63,64]. These findings indicate that delivery and pharmacokinetics, rather than intrinsic lytic activity, often determine clinical performance [64]. Whole phage therapy faces several constraints that limit predictability in clinical settings. Narrow host range remains a central issue and often requires careful matching to the infecting strain, which can drive the use of phage cocktails [65,66]. Pharmacological behavior presents another challenge. Phage pharmacokinetics and pharmacodynamics differ from those of small-molecule antibiotics, and limited clinical-grade data complicate standardized dosing strategies [65].

Host immune responses can further reduce activity. Neutralizing antibodies may develop during treatment, and experimental animal studies have documented rapid neutralization kinetics in infected hosts. Phages can also be inactivated by harsh physiological conditions. Instability in the stomach and upper intestine limits oral dosing when phages are not protected [41,67].

Phage-derived enzymes face distinct limitations. Lysins may show reduced access to peptidoglycan in Gram-negative bacteria due to the outer membrane barrier. Depolymerases can be highly specific for individual capsule types, which restricts coverage across diverse clinical isolates [49,51].

These limitations explain why phage biology alone is often insufficient for reliable performance in complex infections. Biofilms, capsules, immune clearance, and physiological instability all reduce effective exposure at the target site [68].

Importantly, many of these constraints arise from delivery-relevant biological barriers already detailed in Section 2, including biofilm-mediated diffusion limits, capsular shielding, immune clearance, and hostile infection microenvironments. These factors primarily restrict effective exposure and persistence at the infection site rather than intrinsic antibacterial activity.

Accordingly, this section does not restate those barriers but frames them as the mechanistic basis for the delivery-focused solutions discussed in subsequent sections. As discussed in the following section, these constraints provide the rationale for nanotechnology-based platforms designed to improve stability, localization, and access in ESKAPE infections.

## 4. Nanotechnology Platforms for Antibacterial Delivery

**Figure 2:**
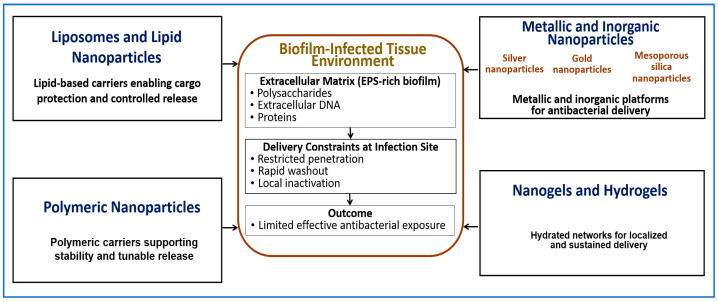
Schematic overview of nanotechnology platforms for antibacterial delivery in biofilm-infected tissues. The central panel highlights key features of the biofilm-infected tissue environment, including the extracellular polymeric matrix and major delivery constraints that limit effective antibacterial exposure, such as restricted penetration, rapid washout, and local inactivation. Surrounding panels summarize representative nanocarrier classes. Liposomes and lipid nanoparticles (LNPs) support cargo protection and controlled release, polymeric NPs enhance stability and tunable release, metallic and inorganic NPs serve as adjustable delivery platforms, and nanogels and hydrogels enable localized and sustained delivery. Overall, the figure illustrates how nano-enabled delivery strategies are designed to address exposure-limiting barriers at infection sites rather than altering intrinsic antibacterial activity.

Nanotechnology-based carriers can improve the in vivo performance of antibacterial agents by protecting sensitive biological cargos, extending residence time at infection sites, and enabling control over release location and timing. These functions are particularly relevant in biofilm-associated infections, where free antibacterial agents often show limited penetration and short exposure [69]. Figure 2 summarizes the major nanotechnology platforms discussed in this section and illustrates how different carrier classes address key exposure barriers in biofilm-associated infections, including limited penetration, rapid washout, and immune-mediated clearance.

Experimental studies demonstrate that nanoparticle penetration into dense bacterial biofilms is strongly size dependent. In *P. aeruginosa* and *Burkholderia* biofilms, particles larger than approximately 100–130 nm penetrate dense biofilm clusters less efficiently, whereas smaller particles show improved access. These values represent practical design constraints rather than fixed thresholds and vary with biofilm composition and matrix density [70]. As discussed in Section 3, many limitations of phage-based and enzyme-based antibacterials arise from delivery constraints rather than from lack of intrinsic antibacterial activity.

Importantly, nano-enabled delivery does not uniformly improve therapeutic performance across all infection contexts [64]. In settings where free antibacterial agents already achieve sufficient exposure, or where infection sites are readily accessible and rapidly cleared, the added formulation complexity and manufacturing cost of nanocarriers may offer limited benefit. These considerations emphasize the need to apply nano-enabled strategies selectively, based on clearly defined exposure barriers rather than as default solutions. Table 1 provides a comparative overview of major nanotechnology platforms, including representative examples, delivery advantages, and key limitations, to support this evaluation.

From a safety perspective, nanoscale size and surface chemistry strongly influence immune recognition, complement activation, and inflammatory responses. Effective carrier design therefore requires balancing delivery efficiency with immunocompatibility [71].

**Table 1:** Representative nanotechnology platforms for antibacterial delivery applications.

Nanotechnology Platform	Representative Antibacterial Cargo	Delivery Rationale	Key Delivery Advantages	Main Limitations/ Constraints	Evidence Level/Current Development Stage	References
Liposomes	Antibiotics; whole phages; phage-derived enzymes	Protect sensitive biological cargos and extend residence time at infection sites	Biocompatible; tunable size and surface properties; improved stability and local exposure	Potential immune activation and tolerability limits depending on dose and formulation; limited stability without surface modification	Primarily in vitro and animal infection models; limited clinical translation (mainly for small-molecule antibiotics)	[72,73]
LNPs	Nucleic acids; CRISPR components; enzymes	Protect nucleic acid cargos and enable controlled intracellular delivery	High cargo protection; tunable composition; established manufacturing frameworks	Potential immune activation; tolerability concerns; pulmonary delivery requires careful dose and formulation control	Primarily in vitro and animal infection models; clinical use established for non-antibacterial nucleic acids	[74,75]
Polymeric NPs (e.g., PLGA)	Whole phages; lysins; antibiotics	Improve stability and enable sustained release in vivo	Biodegradable; controllable release kinetics; adaptable surface modification	Burst release risk; clearance by the mononuclear phagocyte system; scale-up variability	Primarily in vitro and animal infection models	[76,77]
Chitosan-based NPs	Phages; enzymes; antibiotics	Promote interaction with bacterial surfaces and mucosal tissues to enhance local exposure	Mucoadhesive; enhanced local retention; mild formulation conditions	Strong cationic charge may limit deep biofilm penetration; variable solubility under physiological conditions	Primarily in vitro and animal infection models	[78,79]
Metallic NPs (e.g., silver, gold)	Antibiotics; enzymes; combined antimicrobial systems	Serve as delivery carriers or active antimicrobial components	High antimicrobial activity (silver); surface functionalization capacity (gold)	Dose-dependent cytotoxicity; oxidative stress; limited suitability for repeated or pulmonary dosing	Primarily in vitro studies; selected animal infection models	[80,81]
Mesoporous silica NPs	Antibiotics; enzymes	Enable high cargo loading and controlled release	Tunable pore size; structural stability; ease of surface modification	Limited biodegradability; long-term safety considerations	Primarily in vitro and animal infection models	[82]
Nanogels/hydrogels	Phages; lysins; antibiotics	Retain antibacterial agents locally and prolong contact at infection sites	Sustained local release; reduced systemic exposure; suitable for wounds and implanted devices	Limited to accessible infection sites; not suitable for systemic delivery	Primarily in vitro and animal infection models; early translational use in wound care	[83,84]

### 4.1. Liposomes and Lipid Nanoparticles

Liposomes are vesicular systems composed of lipid bilayers that encapsulate hydrophilic cargos within an aqueous core and hydrophobic cargos within the lipid membrane. Liposomal antibiotics have been widely studied to improve local delivery and reduce toxicity in selected clinical settings. Their relevance to biofilm-associated infections is well established, as particle size, surface charge, and membrane fluidity influence interactions with the biofilm matrix and penetration toward embedded bacteria [72]. For biofilm delivery, liposomes are commonly engineered below approximately 100–200 nm, a size range that supports access to biofilm structures and reduces rapid filtration or clearance associated with larger submicron carriers [75,85].

However, liposomes and LNPs are among the nanocarrier classes most frequently associated with complement activation-related pseudoallergy, particularly following intravenous or pulmonary administration. Surface-exposed lipids and insufficient steric shielding contribute to this effect. PEGylation is therefore widely used to reduce opsonization and complement activation, with PEG surface densities of approximately 2–5 mol% PEG-lipid commonly reported to provide mitigation without fully suppressing cellular interactions [86,87].

These safety considerations indicate that lipid-based nanocarriers may offer clear advantages for fragile biological cargos, but may be less suitable for indications requiring repeated systemic dosing or high inhaled doses, where immune activation and tolerability become limiting factors [84,85].

LNPs represent a broader class of lipid-based delivery systems used for small molecules and nucleic acids. Typical formulations include an ionizable lipid, a helper lipid, cholesterol, and a polyethylene glycol lipid [73,88]. Across multiple studies, apparent LNP pKa values in the range of approximately 6.2–6.8 are consistently associated with improved in vivo delivery performance, supporting pKa as a quantitative design parameter rather than a descriptive feature [89,90]. These properties make LNPs attractive for fragile biological cargos that require protection during delivery.

Pulmonary delivery of LNPs requires additional caution. Aerosolized lipid particles can trigger transient inflammatory responses depending on dose, composition, and residual impurities [69,91]. This underscores the need for strict control of formulation purity and inhaled dose [69].

### 4.2. Polymeric NPs and Polymer-Based Carriers

Polymeric NPs are widely used to protect antibacterial agents from degradation and to control release profiles. Many systems rely on biodegradable polymers that degrade into well-tolerated products. Poly(lactic-co-glycolic acid) is among the most extensively studied polymers in drug delivery, with composition, molecular weight, and particle size adjusted to tune release behavior and stability [74].

From an in vivo perspective, particles below approximately 200 nm are more likely to avoid rapid mechanical filtration and show improved systemic persistence. Larger particles are cleared more readily and tend to accumulate in the liver and spleen [75].

Polymeric carriers generally show lower acute immunogenicity than metallic systems. However, surface chemistry remains critical. Cationic polymers have been associated with membrane disruption, complement activation, and inflammatory cytokine release at higher doses. Surface shielding strategies such as PEGylation or zwitterionic coatings are therefore commonly used to reduce nonspecific immune activation [71].

Chitosan is a cationic polysaccharide frequently used to promote interaction with negatively charged bacterial surfaces or mucosal tissues. Modified chitosan-based nanoparticles have been developed to improve solubility and performance under physiological conditions [77]. However, biofilm matrices contain anionic components such as extracellular DNA and polysaccharides. These components can electrostatically trap strongly cationic particles near the biofilm surface. Charge-modulation or charge-switching designs are therefore used to balance penetration with retention [76,84]. Other polymer platforms include PEG-modified systems and hybrid lipid–polymer designs, which aim to combine cargo protection with improved circulation behavior [88].

### 4.3. Metallic and Inorganic NPs

Metallic and inorganic NPs are used either as delivery carriers, as active antimicrobial agents, or as combined systems. Silver (Ag) NPs exhibit broad antimicrobial activity and induce multiple stress responses in bacteria. Their biomedical use requires careful control of particle size and silver ion release, as these properties strongly influence both efficacy and toxicity [79,92]. Smaller AgNPs are generally more reactive but also exhibit narrower therapeutic windows due to increased toxicity [78].

Across nanocarrier classes, metallic NPs are associated with higher risks of oxidative stress, inflammatory signaling, and dose-dependent cytotoxicity. These risks are particularly relevant for pulmonary exposure, where stricter dose limits and justification are required when metallic nanoparticles are used as delivery platforms rather than as active antimicrobials [93].

Gold NPs are widely studied as delivery platforms because they can be readily functionalized with drugs, ligands, or biological molecules. Their biological behavior is strongly influenced by particle size, shape, and surface chemistry [94].

Silica-based NPs, including mesoporous silica NPs, are valued for high loading capacity and ease of surface modification. Their pore structure and surface area support controlled release and targeting in antibacterial applications [80]. Mesoporous materials typically have pore sizes between 2 and 50 nm. Common systems such as MCM-41 often have pore diameters of approximately 2.5–6 nm, which is directly relevant to cargo loading and release [81].

### 4.4. Nanogels and Hydrogels for Local Delivery

Nanogels and hydrogels are hydrated polymer networks that retain antibacterial agents and release them over time. They are primarily designed for local treatment, where high concentrations at the infection site are required and systemic exposure is less desirable. Hydrogel-based delivery supports sustained release and prolonged contact in biofilm-associated infections and is particularly useful in wounds and around implanted devices, where retention and moisture control are key design considerations [82,95].

### 4.5. Biofilm-Targeted and Stimuli-Responsive Nanomaterials

Nanocarriers can be engineered to improve localization and release within biofilm-infected environments. Design objectives include enhanced retention, improved movement through the biofilm matrix, and increased exposure near embedded bacterial cells [83,91]. Consistent with penetration studies, particles near or below approximately 100–130 nm often show improved access to dense biofilm clusters, although optimal values depend on biofilm composition and particle chemistry [70].

Stimuli-responsive systems release cargo in response to infection-associated conditions. Common triggers include acidic pH, enzyme-rich environments, redox conditions, and externally applied stimuli such as light or heat [83]. Biofilm and infected-tissue microenvironments often exhibit pH values in the range of approximately 5.5–6.5. This supports pH-triggered or charge-switching designs as quantitatively justified strategies rather than purely conceptual approaches [84,96].

Biofilm-targeted designs emphasize interactions with extracellular polymeric substances (EPS) and bacterial surfaces. Particle size and surface charge strongly influence diffusion, penetration, and retention [76]. Cationic nanoparticles often bind strongly to anionic biofilm matrices. This can improve retention but may limit deep penetration through a binding-site barrier. Charge modulation or charge switching after penetration can help address this limitation [76,91]. Mechanistically, electrostatic binding to anionic matrix components can improve retention but may also limit deep penetration (“binding-site barrier”), motivating designs that either neutralize ionic trapping or switch charge in situ [76,84]. In many recent systems, targeting and responsiveness are combined to balance penetration, retention, and localized release [83].

### 4.6. Safety and Regulatory Considerations

Nanomedicines face development challenges that differ from those of small-molecule drugs. Regulatory agencies emphasize detailed characterization of physicochemical properties, control of manufacturing variability, and evaluation of how nanoscale features influence distribution and safety.

The United States Food and Drug Administration (U.S. FDA) has issued guidance for drug products that contain nanomaterials, including biological products [97]. The European Medicines Agency (EMA) has published reflection papers and reports addressing evaluation of nanotechnology-based medicinal products, including specific dosage forms such as block copolymer micelles [98]. International harmonization efforts are supported by guidance from the Organization for Economic Co-operation and Development on testing and evaluation of manufactured nanomaterials [99].

### 4.7. Limitations of Nano Drug Delivery Independent of the Antibacterial Cargo

Nanocarriers are not universally advantageous. They may exhibit instability during storage or after administration and can show rapid clearance or unexpected tissue accumulation, depending on size and surface properties. Safety risks are strongly influenced by dose, surface chemistry, dissolution behavior, and aggregation state [100]. Renal filtration is efficient only for very small particles, typically below approximately 5.5 nm. Larger particles avoid renal loss but may be cleared by the mononuclear phagocyte system. Particles above approximately 200 nm are often removed more rapidly and accumulate in the liver and spleen [101].

Manufacturing and scale-up present additional challenges. Batch-to-batch variability can alter particle size distribution, encapsulation efficiency, and release behavior. In antibacterial applications, such variability can change exposure at infection sites and complicate reproducible efficacy. Regulatory guidance therefore emphasizes robust characterization and quality control throughout development [97,98].

Most nano-enabled delivery strategies discussed in this section are supported primarily by in vitro studies and animal infection models. While these studies demonstrate improved stability, retention, or exposure, clinical translation remains limited. Key gaps include standardized dosing metrics, long-term safety data, and controlled human efficacy studies.

## 5. Nanotechnology-Enabled Delivery of Whole Phages

Whole bacteriophages must remain infective from manufacturing through delivery to the infection site. In practice, free phages often lose activity during formulation, storage, and exposure to physiological fluids. These vulnerabilities have driven the development of carrier-based formulations designed to protect phage infectivity and improve local availability at sites of infection [102,103].

Losses of infective phage during processing can reach approximately 1–3 log units, depending on formulation and delivery conditions. Such losses have been documented in aerosolization and drying studies [103,104]. Across published work, three recurring formulation goals emerge. The first is protection from inactivation, including gastric acidity and mechanical stress during aerosolization. The second is extension of residence time at the infection site through controlled release. The third is improvement of effective exposure in environments where diffusion and washout limit the activity of free phages, such as wounds and biofilm-associated infections [102].

### 5.1. Lipid-Based Encapsulation of Whole Phages

Lipid-based encapsulation has been explored as a strategy to reduce phage neutralization and to support delivery in infection models where bacteria persist within host cells. In a study by Singla and colleagues, liposome-entrapped phages retained infective titers after exposure to neutralizing antibodies. In contrast, free phages were rapidly inactivated under the same conditions. The study also reported delivery of entrapped phages into macrophages and a reduction in intracellular *K. pneumoniae* burden [105]. In this model, encapsulation preserved detectable phage activity over timeframes in which free phage titers declined, indicating improved exposure rather than altered phage replication.

Lipid encapsulation has also been evaluated in wound infection models. Chhibber and colleagues examined a liposome-entrapped phage cocktail in a diabetic excision wound infected with *S. aureus*. Improved wound outcomes were reported compared with non-encapsulated phage [106]. The observed benefit correlated with prolonged phage retention at the wound surface, consistent with reduced washout rather than increased intrinsic lytic potency.

Pulmonary delivery further motivates the use of lipid-based systems. A study by Sawant and colleagues reported that liposomal encapsulation reduced phage viability loss during nebulization compared with phage suspension [107]. Longer extracellular retention was also observed in a lung epithelial cell model. Nebulization-associated losses exceeding 1 log unit for unprotected phages have been reported in earlier studies, highlighting the protective effect of lipid encapsulation [103,108].

### 5.2. Polymeric and Composite Encapsulation for Protection and Sustained Availability

Polymer-based encapsulation has been widely investigated to protect phages during oral administration and to extend persistence after delivery. A well-established study developed chitosan–alginate–calcium chloride microspheres for oral delivery of phage Felix O1. Free phages showed high sensitivity to simulated gastric conditions, while encapsulated phages retained infectivity under acidic exposure and were released under intestinal-like conditions [109]. Free phages were rapidly inactivated at gastric pH (<3), while encapsulated phages retained infectivity under the same conditions [109].

More recent studies have applied composite systems to improve in vivo persistence. One report described poly(lactic-co-glycolic acid)–alginate composite microspheres designed to increase phage lifespan after administration. Encapsulated phages were detected in tissues for longer periods than non-encapsulated phages in the same experimental setting [110]. This extended detectability reflects sustained local availability rather than enhanced replication, emphasizing delivery as the primary determinant of performance. These findings indicate that polymeric and composite systems are most often selected when protection from harsh environments is required or when prolonged local availability is needed beyond what free phages can achieve.

### 5.3. Hydrogels and Local Matrices for Topical and Site-Specific Delivery

Hydrogels are commonly used when the infection site is accessible and sustained contact can be maintained, such as in wound infections. These systems retain phages at the surface and support gradual release. This helps counter washout and dilution in exudative environments [111]. Local hydrogel matrices have been shown to maintain high phage concentrations for extended periods, ranging from hours to days. In contrast, free phages are rapidly diluted or removed in dynamic wound environments [111].

Abed and colleagues developed a phage-containing hydrogel for *E. faecalis*-infected wounds and reported controlled release with improved wound outcomes in the tested model [112]. Other studies have described phage-releasing dressings based on three-dimensional fiber structures, with suppression of bacterial growth observed during the release period [113].

Hydrogels have also been engineered to respond to infection-associated cues. Tao and colleagues described an injectable hydrogel that released a dual phage cocktail in response to *P. aeruginosa* infection signals [114]. This approach links phage release kinetics directly to infection-associated signals rather than passive diffusion alone.

### 5.4. Pulmonary Delivery and Aerosol-Related Loss of Phage Activity

Respiratory delivery is an attractive route for treating lung infections, but aerosol generation can substantially reduce viable phage titers. One study quantified infective titer loss after nebulization and showed strong dependence on the delivery setup [104]. Other studies reported structural damage and loss of viability associated with nebulizer type and operating conditions [103,108]. Reported losses typically range from approximately 0.5 to more than 2 log units, directly affecting delivered dose [104,108].

Dry powder inhalation avoids liquid nebulization but introduces stress during drying and storage. Spray-dried phage powders have been shown to retain biological activity when formulation parameters and storage conditions are carefully controlled [115]. A more recent long-term stability study evaluated spray-dried powders active against *P. aeruginosa* and focused on maintaining activity during extended storage [116]. These findings indicate that pulmonary phage products require coordinated optimization of formulation parameters and delivery devices to preserve infectivity from manufacture through administration.

### 5.5. Immune Neutralization and How Encapsulation Can Help

Immune recognition can reduce the activity of free phages, particularly during repeated dosing. Liposome entrapment has been reported to reduce antibody-mediated neutralization in vitro compared with free phages [105]. Encapsulation delays, but does not eliminate, immune-mediated clearance. This highlights modulation rather than avoidance of host immune responses as the realistic goal.

Encapsulation may also alter interactions with phagocytic cells. In the macrophage model reported by Singla and colleagues, liposome-entrapped phages were delivered into macrophages and reduced intracellular bacterial burden [105,117]. This suggests a potential advantage for infections with intracellular components.

### 5.6. Evidence Base and Formulation-Specific Limitations

Across delivery platforms, studies most consistently report improved phage stability, increased tolerance to processing, and prolonged local availability compared with free phages. These outcomes depend strongly on formulation design and infection model. Importantly, improvements are measured as prolonged detectable titers and delayed inactivation rather than increased intrinsic bactericidal activity [102].

Formulation also introduces tradeoffs. Encapsulation can reduce the fraction of immediately available free phages, potentially delaying early antibacterial effects if release is slow. Manufacturing steps can further reduce titers when conditions are not optimized, a recurring issue in aerosolization and drying workflows [103].

Overall, nano-enabled delivery of whole phages aims to improve exposure and persistence without altering core phage biology. This delivery-focused logic provides the foundation for the following sections, which examine nano-enhanced delivery of phage-derived enzymes and comparative strategies across ESKAPE pathogens and infection sites [102,103].

## 6. Nano-Enhanced Delivery of Phage-Derived Enzymes

Phage-derived enzymes act as purified proteins rather than self-replicating particles. Their antibacterial effect depends on reaching the bacterial surface in an active form and remaining stable long enough to exert activity. In vivo, many enzymes lose function because proteins can unfold, aggregate, or be cleared rapidly. Studies of peptidoglycan hydrolases report short serum circulation half-lives, typically on the order of approximately 20–60 min without modification [118]. As a result, therapeutic exposure may be limited even when strong activity is observed [119,120].

Nanoformulation addresses challenges that differ from those of whole-phage delivery. For enzymes, the primary goals are preservation of structure, protection from premature degradation, and control of residence time at the infection site. The following subsections summarize nano-enabled approaches evaluated for lysins, lysostaphin, and other phage-derived antibacterial proteins.

### 6.1. Stability and Nanoformulation of Lysins and Related Enzymes

Phage-derived enzymes such as lysins and lysostaphin are administered as purified proteins. Their activity depends on maintaining structural integrity and achieving access to the bacterial surface. Although many of these enzymes show strong in vitro activity, in vivo effectiveness is often reduced by rapid clearance, unfolding, or aggregation. These processes result in short half-lives and limited exposure at infection sites [119]. Protein instability in the absence of formulation is a well-recognized limitation in protein drug delivery [121].

Nanoformulation is used to mitigate these limitations. Encapsulation or immobilization can reduce exposure to degrading conditions and slow clearance from biological fluids. Reviews of protein delivery describe nanocarriers as tools that protect sensitive proteins and enable controlled release while preserving enzymatic function [122].

Several studies have applied these strategies to lysins. Kaur and colleagues prepared alginate and chitosan nanoparticles loaded with the anti-staphylococcal endolysin LysMR5. The formulation was produced under mild conditions and retained antibacterial activity against staphylococci in the tested system [120,123]. This work demonstrated that polymer-based carriers can preserve enzyme activity while improving stability and handling.

Lysostaphin has also been formulated using biodegradable nanoparticles. A 2024 study reported lysostaphin-loaded poly(lactic-co-glycolic acid) nanoparticles and evaluated activity against multiple *S. aureus* strains. Preserved antibacterial activity following encapsulation was observed, supporting the use of polymeric carriers to stabilize lytic enzymes [124]. Such systems often show extended local retention compared with aqueous enzyme solutions, improving effective exposure near the infection site [121].

These studies illustrate a central principle. For phage-derived enzymes, nanocarriers are used primarily to maintain protein stability and effective exposure at the bacterial surface. Unlike whole phages, enzymes do not replicate. Their therapeutic effect therefore depends on sustained local availability rather than amplification at the infection site [120].

### 6.2. Lysin-Loaded Hydrogels for Local Delivery in Bone and Wound Infections

Hydrogels are frequently selected for local enzyme delivery because they retain proteins at the infection site and enable sustained release. Yao and colleagues described an alginate hydrogel loaded with the chimeric lysin ClyC for treatment of *S. aureus* osteomyelitis. Sustained release and reduced bacterial burden were reported in a mouse model [125]. In this system, the hydrogel maintained detectable enzyme activity for approximately 24–48 h after administration, compared with rapid decline for non-formulated enzyme [120].

Local delivery platforms of this type are particularly useful when systemic administration is limited by rapid clearance or when high local concentrations are required in poorly perfused tissue. These conditions are common in bone and chronic wound infections [122].

### 6.3. Formulation Routes That Require Protection During Processing

Some delivery routes impose additional stress on protein therapeutics. Pulmonary delivery is one example, as drying and aerosolization can reduce enzymatic activity. A 2023 study reported spray-dried powders of the endolysin Cpl1 designed for inhaled delivery. Acceptable aerosol performance and retained antibacterial activity were achieved under optimized conditions [126]. Consistent with the broader pulmonary protein delivery literature, spray-drying can cause substantial activity loss when protective excipients or carriers are absent. These findings highlight the need for formulation strategies that preserve activity during processing, storage, and device-mediated delivery [121,122].

### 6.4. Depolymerases as Antivirulence Enzymes and Delivery-Relevant Considerations

Depolymerases degrade surface polysaccharides such as capsules and weaken defenses that block access to bacterial cells. Studies of *Klebsiella* phage depolymerases show that capsule removal can increase susceptibility to host immune factors and support bacterial clearance in strain-matched settings [127,128].

A 2022 study discussed depolymerases as antibiotic adjuvants and summarized evidence that capsule degradation enhances susceptibility to immune attack and antibacterial treatment [129]. For these enzymes, delivery priorities include maintaining activity in protein-rich infection environments and ensuring effective contact with the capsule or biofilm matrix. Reviews focused on translational development emphasize the need to address stability, dosing, and persistence [130].

### 6.5. Tailocins and Related Phage Tail-like Antibacterials

Tailocins are contractile protein assemblies that resemble phage tails and kill bacteria through receptor-dependent binding and membrane disruption. They act without replication and often display very narrow target spectra [131]. Because tailocins are large and structurally complex, therapeutic use depends on preserving assembly integrity and supporting delivery to the infection site. The current literature emphasizes their potency and specificity, while also noting that additional formulation and delivery development is required to support clinical application [132].

### 6.6. How Enzyme Delivery Differs from Whole-Phage Delivery

Whole phages require preservation of infectivity and access to host bacteria to support replication. Phage-derived enzymes do not replicate. They act directly, and their effect is determined by local concentration and exposure time. Unformulated proteins are often rapidly cleared or degraded in vivo. For peptidoglycan hydrolases, reported half-lives commonly range from minutes to approximately one hour without stabilization [118].

This distinction shifts formulation priorities. For enzymes, the most important outcomes are structural stability, residence time, and access to bacterial targets such as peptidoglycan, capsule, or biofilm matrix [119]. These differences explain why enzyme-focused nanoformulations often favor local delivery platforms, including hydrogels and polymeric particles. Performance is therefore assessed by retained activity and sustained exposure rather than by amplification at the infection site [120,125].

## 7. Nano-Assisted CRISPR Phage and CRISPR Nanoparticle Antibacterials

CRISPR-based antibacterials aim to eliminate bacteria or remove resistance determinants by targeting genetic material with sequence-level specificity. This strategy differs fundamentally from whole-phage therapy and from phage-derived enzymes. It does not rely on phage replication or on protein-mediated cell wall disruption. Instead, its effectiveness depends on successful delivery of the CRISPR system into the target bacterium and on intracellular activity at the intended genetic locus [57,133]. In experimental delivery systems, the overall antibacterial effect is strongly tied to the efficiency of CRISPR delivery into cells rather than the intrinsic catalytic activity of the nuclease itself; limited in vivo delivery efficiency remains a key gap [32]. At present, the vast majority of evidence supporting CRISPR-based antibacterial strategies derives from in vitro studies and small-animal infection models, with no controlled clinical data available [32,57,133].

Early proof-of-concept studies demonstrated that RNA-guided nucleases can be delivered to bacteria to selectively kill strains carrying defined resistance or virulence genes. For example, bacteriophage-based delivery of CRISPR-Cas9 phagemids has produced up to ~2–3 log reductions in bacterial counts in vitro and significant reductions in bacterial burden in animal models when targeting chromosomal genes (e.g., *Shigella* in zebrafish larvae) [32]. Citorik and colleagues used bacteriophage-based delivery to introduce CRISPR constructs targeting specific DNA sequences. They reported sequence-dependent killing and the ability to reshape mixed bacterial populations based on genetic signatures [57]. These studies establish biological feasibility but do not yet define dose and response relationships, durability of effect, or safety profiles relevant to human infection [133,134].

### 7.1. What CRISPR Systems Can Do in Antibacterial Applications

DNA-targeting systems such as Cas9 introduce double-strand breaks in chromosomal DNA and can be bactericidal when repair fails. When the target sequence is located on a plasmid, the outcome can differ. Plasmid cleavage may result in plasmid loss without immediate cell death, which is an important distinction when resistance genes are plasmid-borne [57,61]. Reviews on CRISPR-Cas9 plasmid targeting highlight the potential to cure resistance phenotypes in vitro, although efficient in vivo delivery remains limited [134].

RNA-targeting systems such as Cas13a have been developed to address this limitation. CRISPR-Cas13 systems delivered using phage capsids or related vectors have demonstrated sequence-specific killing after recognition of targeted resistance or virulence genes, with the degree of killing varying with delivery format and target accessibility [135].

Another antibacterial objective is plasmid curing removal of resistance plasmids to restore antibiotic susceptibility rather than direct bacterial killing. Temperate and lytic phages programmed to deliver CRISPR constructs that destroy resistance plasmids have shown promise in vitro and in animal models, though in vivo efficiency remains a key translational challenge [134].

### 7.2. Phage-Based Delivery Formats for CRISPR Cargo

Phages and phage-derived particles are widely used for CRISPR delivery because they efficiently introduce nucleic acids into bacterial cells. Several studies have employed phage-like delivery vehicles to transfer CRISPR components without relying on full therapeutic phage replication [57].

One well-characterized example is a broad-host-range P1-derived phagemid system used to deliver Cas9 constructs targeting chromosomal genes into *E. coli* and *Shigella flexneri*. In this work, a P1 phagemid delivering CRISPR-Cas9 achieved sequence-specific lethality in vitro with ~2–3 log reductions in CFU and significantly improved survival in zebrafish larvae infection models when administered locally [32]. Despite these encouraging animal data, phage-delivered CRISPR systems have not yet progressed to controlled clinical evaluation [133,134].

Non-replicative phage particle delivery systems have also been developed to target resistance plasmids, although quantitative in vivo data for these platforms remain scarce relative to classic antimicrobial models.

### 7.3. Nanoparticle-Assisted CRISPR Delivery in Antibacterial Settings

NPs are widely used to protect nucleic acids and enhance delivery in biomedical applications. In antibacterial CRISPR strategies, a major challenge is achieving intracellular access to bacterial cells while maintaining cargo integrity. A recent study reported lipid nanoparticle-mediated delivery of CRISPR Cas13a for control of bacterial infection, supporting the concept that lipid carriers can function as delivery platforms for antibacterial gene-targeting agents [136]. However, most nanoparticle-assisted CRISPR delivery studies remain limited to in vitro systems or proof-of-concept animal models, and no clinical data are currently available for antibacterial applications [134,136,137].

Most NP-based CRISPR delivery systems have been developed for mammalian cells. However, the underlying delivery principles remain relevant. Reviews describe how lipid, polymeric, and gold-based nanocarriers protect CRISPR components from degradation and influence uptake and biodistribution. These principles inform hybrid strategies in which phage-based delivery enables bacterial entry, while nanomaterials contribute protection, stability, or co-delivery functions [137].

### 7.4. Target Genes and Resistant Subpopulations Relevant to ESKAPE Pathogens

CRISPR-based antibacterials are often discussed in the context of high-priority resistance genes. These include *mecA* in methicillin-resistant *S. aureus* and carbapenemase genes such as *blaKPC*, *blaNDM*, and *blaOXA-48*-like determinants in carbapenem-resistant Enterobacterales. Reviews focused on AMR highlight these genes as frequent targets because of their clinical relevance and frequent plasmid association [133].

Experimental studies have demonstrated gene-selective effects in models that include carbapenem resistance plasmids. Citorik and colleagues reported targeting of resistance determinants in carbapenem-resistant Enterobacteriaceae, including plasmid-encoded examples [57]. Kiga and colleagues described Cas13a-based capsid delivery constructs targeting multiple resistance genes, including several carbapenemase genes and colistin resistance determinants [61].

A practical advantage of this approach is strain-level selectivity. Because targeting is sequence-based, CRISPR antimicrobials can, in principle, eliminate resistant subpopulations while sparing closely related susceptible bacteria in mixed communities, provided that delivery reaches the intended cells. This concept was emphasized in early CRISPR antimicrobial studies that demonstrated genetic-level selection within complex bacterial populations [57]. At the same time, selective pressure may favor emergence of escape variants lacking the target sequence or possessing altered uptake pathways, an issue that remains insufficiently explored in infection models.

### 7.5. Safety, Off-Target Effects, and Translation Challenges

CRISPR-based antibacterials raise safety considerations different from those associated with phages and enzymes. Target selection must minimize unintended effects on off-target bacteria, and delivery systems must be evaluated for risks of horizontal gene transfer and the emergence of escape variants lacking the targeted sequence or blocking uptake. Reviews highlight these risks alongside challenges in regulatory classification and clinical translation [134]. In particular, the potential for horizontal gene transfer of CRISPR components or associated genetic material remains a key unresolved concern, especially in complex microbial communities [133,134].

Regulatory development presents additional challenges. These products may be classified as combination therapeutics that integrate biological vectors with gene editing cargo, requiring clear definitions of potency assays, biodistribution, persistence, and environmental risk management all areas that remain under active discussion in regulatory science [134].

Overall, nano-assisted CRISPR antibacterials represent a precision strategy with significant potential. However, development remains strongly constrained by delivery efficiency, safety control, and product standardization. At present, the absence of clinical data and limited quantitative in vivo performance data remain major barriers to translation, particularly for complex ESKAPE infections [133].

## 8. Comparative Pathogen-Specific Integration Framework

Many existing reviews discuss phage therapy, phage-derived enzymes, nanocarriers, or CRISPR-based antimicrobials as separate topics. This section follows a different logic. It links pathogen-specific biological barriers to the biological modality most likely to be effective. It also outlines delivery design considerations needed to achieve sufficient exposure at the infection site. The focus is on delivery rather than on individual technologies. By organizing evidence around exposure-limiting barriers, this framework goes beyond descriptive comparison and supports structured evaluation of delivery and therapeutic strategies for ESKAPE infections.

**Figure 3:**
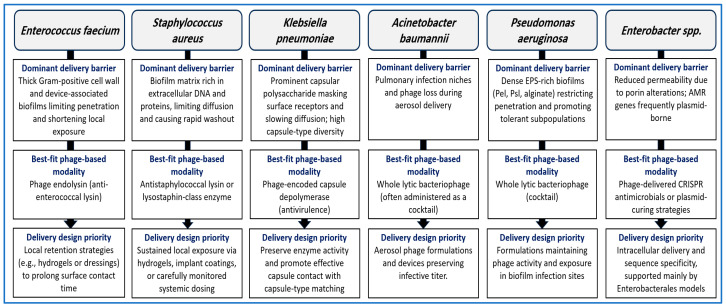
Pathogen-specific schematic linking dominant delivery barriers to phage-based modalities and delivery priorities in ESKAPE infections. For each pathogen, the scheme summarizes the key biological barrier at the infection site, the corresponding phage-based modality, and the delivery design priority required to improve effective exposure.

Figure 3 visualizes the delivery logic developed in this section by mapping dominant pathogen-specific biological barriers to the biological modality and nano-enabled delivery strategy most likely to achieve effective exposure at the infection site. This section links the biological barriers presented by ESKAPE pathogens at infection sites to the phage-based modality most likely to be effective and to the delivery design most appropriate for supporting it. The intent is not to restate antibacterial mechanisms. Instead, the goal is to align dominant barriers with delivery logic using published evidence on pathogen traits and on phage-derived antibacterial tools [19,138,139].

### 8.1. How to Read the Framework

Two questions guide this framework. The first is identification of the dominant barrier that limits effective exposure at the infection site. This barrier may involve a thick Gram-positive cell wall with dense biofilm, a capsule that masks surface receptors, an outer membrane with low permeability, or a biofilm matrix that restricts diffusion [19,140].

The second question is selection of the biological modality that can most directly address that barrier. Lysins and lysostaphin act as purified proteins and are most effective against Gram-positive bacteria, where peptidoglycan is accessible at the cell surface [141]. Depolymerases are best suited to infections in which capsules or polysaccharide layers dominate, although their activity is typically capsule-type specific [127].

Whole bacteriophages remain attractive when local amplification at the infection site is needed. In these cases, delivery must preserve infectivity and support contact with bacteria embedded in biofilms or mucus-rich environments [142]. CRISPR-based antimicrobials are most appropriate when the clinical objective is gene-selective killing or plasmid curing. For these systems, intracellular delivery into bacteria remains the primary limitation [95].

### 8.2. Pathogen-Specific Mapping

In infections caused by *E. faecium*, hospital-adapted lineages frequently form biofilms, particularly on medical devices and tissue surfaces. The surface protein esp has been linked to biofilm-associated traits in clinical isolates, and these infections often depend on prolonged surface colonization rather than rapid planktonic growth [139,143]. In this context, sustained local exposure is more important than achieving high systemic concentrations. A lysin or related cell wall-active enzyme represents a suitable biological modality, provided it is delivered using a local matrix that extends contact time at the infection site.

For *S. aureus*, biofilm structure across many strains is strongly supported by extracellular DNA, which contributes to diffusion limitations and reduced antibacterial exposure in device-associated and tissue-based infections [37,144]. These features explain why systemic delivery often fails to achieve sufficient activity at the target site. A lytic enzyme such as a lysin or lysostaphin is the most appropriate modality, combined with local delivery systems that provide sustained exposure. Although combination with non-specific antibiofilm materials is possible, stable and prolonged enzyme activity at the bacterial surface remains the primary requirement.

In infections involving *K. pneumoniae*, the capsule represents the dominant virulence feature and a major delivery barrier. Capsule diversity is extensive, with genomic analyses identifying numerous *K* loci and substantial serotype variation. This heterogeneity complicates strategies that rely on uniform surface recognition [145]. Phage-encoded depolymerases are particularly relevant in this setting because they remove capsular polysaccharide in a capsule-type-specific manner. This specificity has been documented across multiple phage systems targeting defined capsule types [127,145]. The best-fit modality is therefore a depolymerase delivered in a formulation that preserves enzymatic activity and promotes effective contact with the capsule layer.

*A. baumannii* is a major concern in pneumonia and ventilator-associated infections. Whole-phage therapy has been evaluated in pulmonary infection models, and inhaled administration has been reported in recent clinical and compassionate-use experiences [146]. In this setting, local amplification at the infection site can be advantageous, provided that infectivity is preserved during delivery. Whole bacteriophages represent the best-fit modality, paired with aerosol-compatible formulations that protect phage activity during processing and administration.

Biofilm-associated tolerance in *P. aeruginosa* is closely linked to the extracellular matrix. Multiple studies identify alginate, Psl, and Pel as key polysaccharides, with Psl playing a central role in early protection against antibiotics during biofilm development [138,140,147]. These matrix components limit diffusion and reduce effective exposure of antibacterial agents. Whole bacteriophages are well suited when local amplification is desired, but successful application requires delivery designs that enhance penetration into the biofilm and prolong residence within the matrix environment.

For *Enterobacter* species, particularly the *Enterobacter cloacae* complex, reduced permeability caused by porin alterations is a recurring contributor to carbapenem resistance. Decreased expression or loss of outer membrane proteins such as OmpC and OmpF has been reported to limit entry of therapeutic agents and reduce intracellular exposure [19,148]. In this context, CRISPR-based systems offer a practical advantage through gene-level selectivity, including targeting of plasmid-borne resistance determinants. Phagemid-based capsid systems have been reported for delivery of CRISPR Cas13a cargo with sequence-specific antibacterial effects [95]. CRISPR-based targeting therefore represents the best-fit modality when the primary goal is removal of resistance genes or selective pressure against resistant subpopulations, provided that efficient delivery into target bacteria can be achieved.

### 8.3. Comparative Table for Decision Making

Table 2 integrates the pathogen-specific reasoning developed in this section into a structured decision-support framework. It links dominant biological barriers presented by each ESKAPE pathogen to the biological modality and type of nano-enabled delivery most likely to improve exposure at infection sites. The table does not rank therapeutic options or prescribe treatment choices, but instead supports rational, context-dependent decision making.

Figure 3 provides a visual summary of the pathogen-specific framework outlined in Table 1, illustrating how dominant biological barriers guide selection of phage-based modalities and delivery design priorities across ESKAPE pathogens.

### 8.4. Practical Takeaway

Across ESKAPE pathogens, the most important design decision is not the choice of nanocarrier in isolation, but how the biological modality is matched to the dominant barrier limiting exposure at the infection site. Capsular barriers favor depolymerases. Gram-positive biofilm infections are best addressed with lysins delivered in systems that support local retention. Pulmonary infection niches favor whole-phage formulations that remain stable during aerosol delivery. In contrast, infections driven by reduced permeability or gene-level AMR are more appropriately addressed with CRISPR-based systems capable of selective genetic targeting, provided that efficient delivery to the target bacteria can be achieved [95,127,141].

## 9. Translational Challenges and Clinical Pathways

Nano-enabled phage platforms combine a biological antibacterial agent with a material carrier. This integration can improve exposure at infection sites but also increases development complexity. Successful translation requires reproducible manufacturing, clearly defined quality attributes, and clinical pathways that regulators can evaluate using existing frameworks for biological and nanomedicine products [97,98,153]. Despite extensive preclinical progress, robust clinical evidence for nano-formulated phage or phage-derived products remains limited. Most human data involve non-nano phage preparations or local formulations. As a result, potential clinical benefits of nano-enabled systems should be interpreted as delivery-driven hypotheses supported by preclinical evidence rather than as established clinical outcomes.

The challenges discussed in this section build on the delivery-relevant biological barriers outlined in Section 2 and the limitations of unformulated phage therapies summarized in Section 3.7. Rather than restating those constraints, this section focuses on how they affect manufacturing, clinical pharmacology, trial design, and regulatory evaluation. Importantly, while clinical phage therapy experience is increasing, controlled human studies of nano-formulated phage products remain scarce [63,64,154].

### 9.1. Manufacturing and Scale-Up of Nano-Phage Systems

Clinical translation begins with consistent manufacturing. Phage products must be produced under quality systems that control raw materials, process conditions, and contamination risks. Analyses focused on Good Manufacturing Practice describe how phage production can align with existing medicinal product frameworks, while still requiring phage-specific controls [153].

Hybrid formulations introduce additional complexity. Phages or enzymes must remain active during encapsulation and downstream processing. Scale-up alters shear forces, temperature profiles, and residence times, all of which can affect infective titer or enzymatic activity [153,155]. Purification is a central constraint. Injectable phage products require strict control of bacterial impurities and endotoxins. Reviews of translational phage therapy consistently identify purification and contaminant control as major manufacturing priorities [155].

### 9.2. Quality Control, Stability, and Storage

Quality control programs must confirm identity, potency, and purity. For phages, potency is commonly assessed by infective titer, but additional attributes are relevant. These include genome integrity, host range consistency, and absence of unwanted genes. Manufacturing-focused reviews emphasize sequencing-based approaches for identity confirmation and impurity assessment [153].

Stability is a persistent challenge. Hybrid products must maintain performance as both biological entities and nanomaterial systems. U.S. FDA guidance on nanomaterial-containing drug products highlights the need to define critical physicochemical properties and to evaluate how nanoscale attributes influence safety and performance over time [97].

### 9.3. Immunogenicity and Host Compatibility

Host immune responses can reduce phage exposure, particularly during repeat dosing. A controlled animal study reported reduced plasma phage titers following repeated intravenous administration, accompanied by innate immune activation [156]. Human-relevant data are also emerging. Berkson and colleagues reported that phage-specific immune responses reduced phage-mediated effects and documented induction of neutralizing antibodies after treatment [157].

For hybrid systems, compatibility considerations extend to the carrier. Nanomaterials can alter circulation time, tissue distribution, and immune recognition of the biological cargo. Regulatory agencies therefore expect assessment of immunotoxicology and biodistribution when nanomaterials are part of the final product [97,98].

### 9.4. Regulatory Classification and Approval Pathways

Regulatory classification remains challenging for products that combine biological agents with nanomaterials. The U.S. FDA has issued guidance addressing characterization, risk assessment, and control strategies for drug products that contain nanomaterials, including biological products [97].

In Europe, the European Medicines Agency has published reflection papers and reports on evaluation of nanomedicines, including guidance for specific dosage forms such as block copolymer micelles [98]. At the international level, the Organization for Economic Co-operation and Development supports harmonized safety assessment of manufactured nanomaterials and adaptation of testing guidelines to nanoscale properties [99].

Regulatory pathways for phage therapy remain heterogeneous across regions. Reviews of global phage regulation describe variability in national frameworks and ongoing challenges in integrating personalized phage approaches into conventional medicinal product pathways [158].

### 9.5. Clinical Development and Trial Design Challenges

Clinical development must account for pathogen specificity and adaptive treatment strategies. These features complicate patient enrollment, standardization, and comparability across sites. Reports of clinical experience highlight practical challenges, including isolate matching, production timelines, and endpoint selection for complex infections [16].

Endpoint selection requires particular care. Microbiological clearance is important, but clinical outcomes are influenced by surgical intervention, source control, and host factors. Early case series of intravenous phage therapy demonstrated feasibility and safety while identifying design considerations relevant to future trials [16].

Controlled human data illustrate both promise and current limitations. A randomized trial in chronic otitis caused by antibiotic-resistant *P. aeruginosa* supported safety and suggested efficacy [63]. In contrast, an oral randomized trial in children with acute diarrhea did not show clinical benefit despite phage recovery [154]. In the PHAGOBURN phase 1/2 trial for burn wound infections, outcomes were limited by practical PK/PD constraints, including delivered active dose, local inactivation, and susceptibility dynamics [64].

These findings reinforce that effective exposure at the infection site, rather than nominal administered dose, often determines outcome. For nano-enabled phage products, trial design must therefore define dose at two levels: the biological dose and the nanomaterial-associated dose. Handling constraints and stability at the point of care must also be addressed [97].

A key translational gap arises from differences between animal models and human infections. Murine models often involve short time courses and controlled inocula, whereas human ESKAPE infections are heterogeneous and frequently involve biofilms, device-associated niches, and variable immune status. These factors can substantially alter phage and nanocarrier PK/PD [159,160,161].

For phage–nanocarrier conjugates, PK/PD complexity increases further. Clinical performance depends on carrier biodistribution, release kinetics, and density-dependent phage dynamics at the infection site. Dose–exposure relationships therefore cannot be inferred from administered phage titer alone [66,159,160].

### 9.6. Ethical, Logistical, and Access Considerations

Personalized phage approaches raise access and equity concerns. These therapies often require rapid isolate shipment, susceptibility testing, and timely product release. Reviews consistently identify logistics and standardization as barriers to broader deployment [16]. Biosafety and stewardship are also essential. Phage products must be free of unwanted genes and bacterial contaminants. Quality frameworks therefore include genomic characterization and impurity testing as ethical requirements in addition to technical ones [155].

### 9.7. Toward a Translational Roadmap

A practical translational pathway integrates steps often considered separately. The first is definition of the target indication and infection niche. The second is identification of measurable quality attributes for both the biological cargo and the nanocarrier. The third is establishment of manufacturing controls that preserve activity during scale-up. The fourth is alignment of safety packages with nanomedicine expectations and phage-specific risks. Existing regulatory guidance provides structure for this process, even as product-specific standards continue to evolve [97].

This roadmap integrates the biological barriers described in Section 2 and the delivery limitations summarized in Section 3.7 into clinical development logic rather than treating them as preclinical concerns. Bridging the gap between animal models and human infections will require integrated PK/PD frameworks, standardized reporting of exposure and clearance, and carefully designed early-phase trials that explicitly account for delivery-mediated effects [162,163].

Real-world datasets demonstrate feasibility but also reinforce the need for controlled trials in well-defined clinical niches. Reports of large personalized phage therapy cohorts highlight implementation potential but do not substitute for randomized evidence, particularly for nano-enabled products where clinical data remain limited [164].

## 10. Future Directions and Emerging Frontiers

Future progress in nano-enabled phage therapeutics will depend on improved control of exposure at infection sites and more precise matching of biological modalities to patient-specific pathogens. The recent literature points to several converging directions that could increase reliability and support translation into clinical practice, particularly when delivery design, biological specificity, and clinical implementation are considered together [165,166].

### 10.1. Smart, Responsive, and Immune-Aware Delivery Systems

Stimuli-responsive nanomaterials are being developed to release antibacterial payloads under conditions commonly associated with infected tissue. These conditions include acidic microenvironments, enzyme-rich niches, and redox changes linked to inflammation. Such systems aim to enhance local release while limiting exposure in healthy tissue. Reviews focused on biofilm-associated infections describe responsive carriers as a practical approach to improve penetration and antibacterial availability within biofilm-protected regions [83,167].

These strategies are particularly relevant for biological cargos that are more fragile than small-molecule antibiotics. Bacteriophages, lysins, and CRISPR components can lose activity when exposed to heat, shear stress, or unfavorable pH. Responsive release may help preserve function by reducing time spent in harsh environments and by concentrating release where bacteria are present [83,167].

Immune interactions are also becoming a primary design consideration. Host immune responses can shape phage exposure during repeated dosing, and immune recognition has been identified as a factor that can limit sustained efficacy [165,167]. Future delivery systems may therefore be optimized to reduce rapid neutralization, limit inflammatory responses driven by impurities, and maintain exposure within inflamed tissue. These objectives align with broader trends in nanomedicine that emphasize immunotoxicology and control of biodistribution.

### 10.2. Data-Guided Matching and Precision Design

Host range remains a central limitation for phage-based therapies. Machine learning and deep learning approaches are increasingly applied to predict phage–bacterium interactions using genomic and phenotypic data. Recent studies and reviews describe how these tools can accelerate phage selection and prioritize candidates for experimental validation [168].

This data-guided logic can extend beyond phage selection to delivery design. Genomic and phenotypic information describing bacterial surfaces, resistance determinants, and biofilm traits can inform carrier choice and formulation strategy. For example, matrix-rich biofilms may favor delivery systems that enhance residence time, whereas capsule-dominated strains may favor depolymerase-centered approaches. Future decision-support tools are likely to link bacterial genotype and phenotype to a recommended biological modality and a limited set of compatible delivery formats [83,169].

### 10.3. Personalized, Adaptive, and Microbiome-Preserving Therapies

Personalized phage therapy is moving from isolated reports toward more structured clinical use in selected regions. A large retrospective analysis of one hundred consecutive personalized cases reported outcomes across multiple hospitals and infection types, providing insight into real-world implementation [164]. More recent compassionate-use studies have evaluated personalized inhaled phage therapy for cystic fibrosis patients with MDR or pan-drug-resistant *Pseudomonas* infection, illustrating how phages can be selected for individual patients and delivered through the airway route [170].

Nano-enabled delivery may strengthen personalization by improving formulation stability during storage and administration and by extending residence time at infection sites. These effects could reduce dosing frequency and facilitate rapid adjustment when resistance patterns or clinical response change [165].

An additional advantage of phage-based strategies is their narrow targeting compared with broad-spectrum antibiotics. Reviews of clinical experience emphasize the potential to focus treatment on the causative strain while limiting disruption of commensal microbiota [171]. Local delivery formats, controlled release, and surface-targeted designs may further reduce exposure of non-target microbial communities. This consideration is particularly important for high-risk patients, in whom microbiome disruption can contribute to secondary infections or delayed recovery [167].

### 10.4. Theranostic and Integrated Clinical Platforms

Theranostic platforms aim to combine diagnosis and therapy within a single system. In infection management, nanomaterials can support pathogen detection, image-guided localization, and antibacterial delivery in parallel. Recent reviews describe nanotheranostic approaches for infection control and highlight how integration of imaging and therapy may improve treatment timing and targeting [17,172].

For phage-based biologics, theranostic designs could address practical questions during treatment. These include whether a formulation reaches the intended infection niche, how long it persists, and whether bacterial burden is changing over time. Access to this information could support dose adjustment and early switching when response is limited [17,172].

Overall, the most impactful advances are likely to arise from integrated systems that align three elements. The first is selection of a biological modality matched to the dominant pathogen barrier. The second is a delivery format that preserves activity and improves exposure at the infection site. The third is an implementation pathway that supports rapid matching, quality control, and patient-specific adaptation. Progress in personalized practice and predictive modeling suggests that this integrated direction is becoming increasingly feasible [164,169].

**Figure 4:**
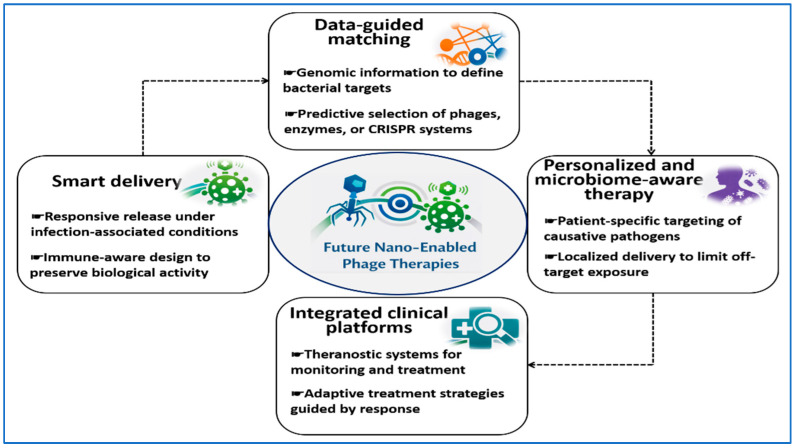
Conceptual roadmap for future nano-enabled phage therapies. The figure summarizes converging directions that may shape the next phase of nano-enabled phage and phage-derived antibacterial development. Smart delivery strategies emphasize infection-responsive release and immune-aware design to preserve biological activity at infection sites. Data-guided matching integrates genomic and phenotypic information to support predictive selection of phages, enzymes, or CRISPR-based modalities. Personalized and microbiome-aware approaches focus on patient-specific targeting and localized delivery to limit off-target exposure. Integrated clinical platforms combine therapeutic delivery with monitoring and adaptive treatment strategies, supporting iterative optimization during clinical use.

These emerging directions are summarized conceptually in Figure 4, which illustrates how delivery design, data-guided matching, personalization, and integrated clinical platforms may converge to support more reliable and adaptable nano-enabled phage therapies.

## 11. Conclusions

ESKAPE pathogens drive antimicrobial resistance through biofilms, surface shielding, reduced permeability, and rapid genetic adaptation. These features primarily limit effective exposure at infection sites, rather than the intrinsic activity of antibacterial agents. Phages, phage-derived enzymes, and CRISPR-based antimicrobials offer selective alternatives to conventional antibiotics, but their performance in vivo depends on how efficiently they reach, persist, and interact with bacteria in complex infection environments. This review highlights three practical conclusions. First, no single phage-based modality is universally effective. Therapeutic success depends on matching the biological agent to the dominant exposure-limiting barrier. Second, delivery design is a primary determinant of outcome. Nano-enabled systems are most useful when they directly address defined constraints such as biofilm penetration, capsular shielding, immune clearance, or rapid washout. Third, a pathogen-aware framework provides a rational basis for selecting the appropriate combination of modality, carrier, and delivery route, rather than treating nanotechnology or phage therapy as standalone solutions. Translation of nano-enabled phage-based therapeutics will be accelerated by progress in several priority areas. These include robust and scalable GMP manufacturing workflows for hybrid biological–nanomaterial products; standardized assays for stability, immunogenicity, and biodistribution; harmonized regulatory pathways for combination products; and controlled clinical trials focused on well-defined infection niches, such as chronic wounds, device-associated infections, and cystic fibrosis–related lung infections. Addressing these priorities will be essential to move nano-enabled phage and phage-derived therapeutics from experimental systems toward reliable clinical interventions for multidrug-resistant ESKAPE infections.

## Figures and Tables

**Table 2:** Pathogen-specific integration of biological modality and delivery strategy.

ESKAPE Pathogen	Dominant Delivery Barrier at Infection Site	Best-Fit Phage-Based Modality	Delivery Design Priority	Key Limitations/Constraints	References
*E. faecium*	Thick Gram-positive cell wall; device-associated biofilms limiting penetration and shortening local exposure	Phage endolysin (anti-enterococcal lysin)	Local retention (e.g., hydrogels or dressings) to prolong surface contact time	Activity has been shown mainly in laboratory studies; in practice, effectiveness depends on keeping the agent at the infection site long enough, as rapid diffusion or clearance can reduce contact time	[149]
*S. aureus* (incl. MRSA)	Biofilm matrix (often eDNA/protein-rich) limiting diffusion and causing rapid washout	Antistaphylococcal lysin/lysostaphin-class enzyme	Sustained local exposure (hydrogel or implant coating) or monitored systemic dosing	Antibacterial activity depends on maintaining sufficient enzyme levels at the infection site; in biofilm-associated infections, poor diffusion and rapid washout can reduce effectiveness unless contact time is prolonged	[150]
*K. pneumoniae*	Prominent capsule masking receptors; slowed diffusion; high capsule-type diversity	Phage-encoded capsule depolymerase (antivirulence) ± matched lytic phage	Maintain enzyme activity and promote capsule contact; capsule-type matching	Activity is capsule-type specific; effectiveness depends on matching the depolymerase to the correct capsular serotype, which limits coverage across diverse clinical isolates	[127]
*A. baumannii*	Outer membrane barrier and pulmonary infection niches, with loss of viable phage during aerosol generation and delivery	Whole lytic bacteriophage (often administered as a cocktail).	Aerosol-compatible formulation and delivery device that preserves infective phage titer during nebulization and pulmonary administration	Phage activity can be reduced during aerosolization and pulmonary delivery; effectiveness depends on preserving viable phage titers and matching phages to the infecting strain	[16]
*P. aeruginosa*	Dense EPS-rich biofilms (Pel, Psl, alginate) restricting penetration and promoting tolerant subpopulations	Whole lytic bacteriophage (cocktail)	Formulations and delivery approaches maintaining phage activity and sufficient exposure within biofilm-associated infection sites	Dense biofilm matrices can limit phage penetration; effective activity depends on sufficient contact with biofilm-embedded cells and may require prolonged or repeated exposure	[151]
*Enterobacter* spp.	Reduced permeability due to porin alterations; antimicrobial resistance genes frequently plasmid-borne	CRISPR-based antimicrobial or plasmid-curing strategy delivered by phage-derived or phage-like vectors	Maximize intracellular delivery efficiency and verify sequence-specific activity; currently best supported by Enterobacterales models rather than direct clinical *Enterobacter* data	Effectiveness depends on getting the CRISPR system into bacterial cells; current evidence comes mainly from laboratory and animal studies, with limited direct clinical data for *Enterobacter* infections	[57,152]

## Data Availability

No new data were created or analyzed in this study.
